# Important Metabolites in Maintaining Folate Cycle, Homocysteine, and Polyamine Metabolism Associated with Ranibizumab Treatment in Cultured Human Tenon’s Fibroblasts

**DOI:** 10.3390/biom9060243

**Published:** 2019-06-22

**Authors:** Siti Munirah Md Noh, Siti Hamimah Sheikh Abdul Kadir, Sushil Vasudevan

**Affiliations:** 1 Faculty of Medicine, Universiti Teknologi MARA (UiTM), Cawangan Sungai Buloh, Selangor 47000, Malaysia; sitih587@salam.uitm.edu.my (S.H.S.A.K.); skvasudevan80@gmail.com (S.V.); 2 University of Malaya Centre for Innovation and Commercialization (UMCIC), University of Malaya, Kuala Lumpur 50603, Malaysia; 3 Institute of Medical Molecular Biotechnology, Faculty of Medicine, Universiti Teknologi MARA (UiTM), Cawangan Sungai Buloh, Selangor 47000, Malaysia

**Keywords:** anti-VEGF, ranibizumab, trabeculectomy

## Abstract

The anti-fibrotic properties of ranibizumab have been well documented. As an antagonist to vascular endothelial growth factor (VEGF), ranibizumab works by binding and neutralizing all active VEGF-A, thus limiting progressive cell growth and proliferation. Ranibizumab application in ocular diseases has shown remarkable desired effects; however, to date, its antifibrotic mechanism is not well understood. In this study, we identified metabolic changes in ranibizumab-treated human Tenon’s fibroblasts (HTFs). Cultured HTFs were treated for 48 h with 0.5 mg/mL of ranibizumab and 0.5 mg/mL control IgG antibody which serves as a negative control. Samples from each group were injected into Agilent 6520 Q-TOF liquid chromatography/mass spectrometer (LC/MS) system to establish the metabolite expression in both ranibizumab treated cells and control group. Data obtained was analyzed using Agilent Mass Hunter Qualitative Analysis software to identify the most regulated metabolite following ranibizumab treatment. At *p*-value < 0.01 with the cut off value of two-fold change, 31 identified metabolites were found to be significantly upregulated in ranibizumab-treated group, with six of the mostly upregulated having insignificant role in fibroblast cell cycle and wound healing regulations. Meanwhile, 121 identified metabolites that were downregulated, and seven of the mostly downregulated are significantly involved in cell cycle and proliferation. Our findings suggest that ranibizumab abrogates the tissue scarring and wound healing process by regulating the expression of metabolites associated with fibrotic activity. In particular, we found that vitamin Bs are important in maintaining normal folate cycle, nucleotide synthesis, and homocysteine and spermidine metabolism. This study provides an insight into ranibizumab’s mechanism of action in HTFs from the perspective of metabolomics.

## 1. Introduction

The application of anti-VEGF has been shown to significantly suppresses neovascularization and fibrosis [1], which are the imperative hallmarks of failed trabeculectomy. Trabeculectomy is a gold standard procedure that is performed when medical and laser intervention fail to maintain the ideal intraocular pressure (IOP) in glaucoma patients [2]. Introduced by Cairns [3], this technique has been shown to effectively reduce the IOP by creating a conjunctiva pocket, an alternative pathway for aqueous humor to escapes from the anterior chamber of the eyes by bypassing the conventional outflow pathways [4,5]. Unlike most surgeries, a completely healed incision at the wound site is unfavorable in trabeculectomy. HTFs are the main effector cells in the initiation and mediation of wound healing and fibrotic scar formation after trabeculectomy [2,6]. Traditional antimetabolites such as mitomycin C (MMC) and 5-Flurouracil (5-FU) are used in trabeculectomy to minimize wound healing and scar formation; however, their application is associated with unwanted post-operational complications including central vision loss, cystic blebs, and hypotony maculopathy due to their nonspecific mechanisms [7,8,9,10,11,12].

Wound healing is a complex cascade that involves various growth factors, chemokines, and cytokines, with one of its best observed part is the creation of a new capillary bed via angiogenesis. The onset of angiogenesis is highly regulated by the numbers of soluble factors, most conspicuously VEGF [13]. Ophthalmic researchers have been focusing on the role of VEGF in ocular angiogenesis, where significant levels of VEGF has been found in the vitreous of diabetic retinopathy and the aqueous humor of rubeotic glaucoma patients [14,15]. There are five members of VEGF found in mammal; however, several studies have shown that VEGF-A is the most dominant proangiogenic factor in healing wounds [13,16]. VEGF-A belongs to a family of mitogenic glycoproteins that promote angiogenesis by the activation of cell surface VEGF receptors present in endothelial and mural cells via a tyrosine kinase (TK) signaling pathway [17]. Therefore, a major focus has been directed to the role of anti-VEGF-A in ocular angiogenesis and its potential use as a wound modulator in trabeculectomy [18,19,20,21]. 

At present, there are two anti-VEGFs currently used as an adjunct in trabeculectomy—bevacizumab and ranibizumab. Kletner et al. has discussed the efficacy of bevacizumab and ranibizumab, and both anti-VEGFs have been shown to exert similar potency in neutralizing VEGF at their clinical dose [22]. Bevacizumab is the first anti-VEGF approved by the US Food and Drug Administration (FDA) for human cancer therapy, particularly for colorectal cancer [23]. Due to its potential as an antiangiogenic and antiproliferative agent, bevacizumab has been used “off record” in trabeculectomy as part of an antiscarring strategy [20,24,25]. Surprisingly, although bevacizumab acts as an anti-VEGF, a recent in vitro study revealed that bevacizumab antiproliferative and cell-death inducing effects on HTFs are not dependent on VEGF inhibition [26]. It was speculated that the secreted VEGF promotes angiogenesis in wound healing, rather than directly enhancing HTFs proliferation. Ranibizumab is a recombinant, humanized monoclonal antibody antigen-binding fragment (Fab) directed against VEGF-A which has been approved by FDA to be used in the eye for the treatment of wet age macular degeneration (AMD) [27,28]. It acts against all VEGF-A isoforms and was found to be more effective in treating neovascular AMD than pegaptanib [29]. Intravitreal application of ranibizumab had a pronounced effect in trabeculectomy, where it resulted in more diffuse bleb with less vascularity, thus maintaining the desired IOP [30]. Interestingly, in vitro studies on cultured HTFs have highlighted the potency of ranibizumab in suppressing cell viability and in interfering the synthesis of collagen Type 1, fibronectin, transforming growth factor (TGF)-*β*1, and TGF-*β*2 [31,32]. However, the definite mechanism of actions of ranibizumab on these common proteins are that involved in tissue scarring are not well understood; thus, further investigation is warranted to truly understand how ranibizumab affects HTF’s activity at the metabolic level. 

As its names indicates, metabolomics is a quantitative measurement of small molecules which are the intermediate or end product of various cellular processes [33,34]. In the last century, there has been rapid development of the separation and detection techniques of small molecules. These methods comprise gas chromatography (GC) and liquid chromatography (LC) for separation and mass spectrophotometry for detection. This technology has led to the discovery of numerous metabolites which represent the biochemical pathways of carbohydrates, fats, proteins, nucleic acids, or xenobiotics [35,36] and is shown to be an effective tool for disease diagnosis [37,38,39], biomarker screening [40,41], and characterization of biological pathways [42,43]. More interestingly, metabolomics has also become a powerful platform to investigate alteration of metabolites in specific cells following drug therapy. However, a systematic metabolomics analysis on HTFs following ranibizumab treatment has not yet been carried out. Therefore, this study aims to establish the metabolite profile of ranibizumab-treated HTFs using untargeted metabolomics analysis. This piece of novel data is important to recognize the effects of ranibizumab on the expression of these metabolites, and their roles in modulating HTF activity. These new findings will offer a better approach in wound modulation strategies, thus increasing the success rate of trabeculectomy. 

## 2. Material and Methods

### 2.1. Human Tenon’s Explant Culture

Primary HTFs were propagated from Tenon’s capsules, which were explanted from patients with primary open-angle glaucoma (POAG) who were undergoing trabeculectomy [44]. Subject inclusion criteria are male and female with an age range of 18 to 65, while subject exclusion criteria are patients under topical anti-glaucomatous drugs with a history of inflammatory condition such as uveitis, episcleritis, and scleritis. This study adhered to the ethical approvals from Universiti Teknologi MARA Ethics Committee Board; 600-RMI (5/1/6). National Medical Research Register (NMRR) granted Ethical Approval to carry out the study within facilities (Research ID:10601). 

Patients gave their written informed consent. Each patient Tenon’s capsule was cultured individually and the primary HTFs were harvested as an expansion culture and propagated in Roswell Park Memorial Institution (RPMI) culture media (Gibco; Life Technology Grand Island, NY). The culture media were supplemented with fetal bovine serum (FBS), 20% of the final volume (Gibco, Life Technology), penicillin 100,000 U/I, and streptomycin 10 mL/L (Gibco). The cells were maintained at 37 °C in 5% CO in a humidified atmosphere. Only HTFs between the third and sixth passages were used for all experiments. Cells at the higher passage indicated altered morphology compared to the ones at the lower passage, and this might affect the response to treatment. Purity of HTFs were verified by anti-vimentin antibody as presented in Supplementary Figure S1. 

### 2.2. Human Tenon’s Fibroblast Culture

HTFs were seeded in six-well plates at a density of 3 × 10^5^ cells/well in complete RPMI culture media supplemented with 5% FBS and incubated for 24 h in humidified environment at 37 °C with 5% CO_2_. Then, the culture media was replaced with serum-free media and incubated further for 24 h in the same environment. Subsequently, the HTFs monolayer was treated with 0.5 mg/mL of ranibizumab Novartis (Basel, Switzerland) as described by Noh et al. [32], which has been diluted in serum-free media and incubated for 48 h. The wells that served as the control were treated with control IgG antibody (Invitrogen, Waltham, MA, USA) with the same concentration. Once the incubation was completed, supernatants were discarded. The following steps were performed on ice to preserve the integrity of the metabolites. Phosphate buffer saline (PBS) was added to each well to wash the HTFs monolayer and later discarded. Then, PBS was added again into each well and the HTFs monolayer was scrapped off using cell scrapper. The cell extract was then transferred into centrifuge tube and centrifuged at 10,000× *g* for 5 min at 4 °C by using Eppendorf Centrifuge 5804R (Eppendorf, Hauppauge, NY, USA). These washing steps were repeated three times. Upon completion of the final washing step, the supernatant was removed and the cell pellet was quickly stored in liquid nitrogen to snap freeze the metabolites that were present in the samples. 

### 2.3. Metabolite Extraction

The cell pellet stored in liquid nitrogen was thawed at room temperature and then transferred into a microcentrifuge tube. Distilled water (dH_2_O) with pH 10 was added into each tube followed by the addition of absolute methanol (Sigma Aldrich, Saint Louis, MO, USA). The cell suspension was agitated in a tube shaker for 20 min at 11,000× *g*. Then, the cell suspension was sonicated for one minute at 0 °C. Subsequently, absolute chloroform (Merk Milipore, Burlington, MA, USA) was added into the cell suspension, resuspended, and vortexed. The cell suspension was sonicated again for one minute at 0 °C. The suspension was transferred into a new microcentrifuge tube and centrifuged for 20 min at 11,000× *g* with 4 °C. After centrifugation, different layers were separated. Polar metabolites were extracted in upper layer and the nonpolar metabolites were extracted in the lower layer. The upper layer and lower layer were collected and transferred to new 2 mL centrifuge tube and mixed thoroughly. The samples were then dried at 0 °C for 120 min using an Eppendorf Concentrator Plus and were vortex-mixed for one minute before they were finally transfer into vials for LC/MS analysis. 

### 2.4. LC/MS Q-TOF Analysis and Data Processing

A 1 µL sample was injected splitless into an Agilent 6520 Q-TOF liquid chromatography/mass spectrometer (LC/MS) system (Agilent Technologies, Santa Clara, CA, USA) equipped with dual-ESI source. Zobrax Eclips Plus C18-ID of 1.8 µm particle size and 2.1 × 100 mm column dimension was used. The temperature was maintained at 40 °C during the run.

The mobile phase consisted of two main components: (A) 0.1% formic acid in water and (B) 0.1% formic acid in acetonitrile. The flow rate was set at 0.25 mL/minutes and the injection volume was 2 µL. A linear gradient was developed over 36 min from 5% to 95% of mobile phase (B). Total run time was 48 min for each analysis. ESI Source setting was as follows: V Cap 4000 V, skimmer 65 V, and fragmentor 125 V. The mass spectral acquisition ranger was set from 50 to 1400 mass to charge ratio (m/z). The nebulizer was set at 45 pounds per square gauge (psig) and the nitrogen drying was set at flow rate of 12 L/minutes. Drying gas temperature was maintained at 350 °C. Data was acquired at rate of 2.5 spectra/seconds with a stored range of m/z 50–1000. Internal reference ions were used to correct mass accuracy. Autocalibration parameters were chosen to average ten scans and reference mass correction was enabled throughout the run. In order to obtain profiles containing as many as possible of this untargeted study, the generic setting was chosen for both LC separation and MS analysis. Continuous internal calibration was done to assure the desired mass accuracy of the recorded ions.

Agilent Mass Hunter Qualitative Analysis Software B.05.00 (Agilent Technologies, Santa Clara, CA, USA, 2011) was used to process the data after it had been collected by Agilent Mass Hunter Workstation Data Acquisition Software B.02.01 (Agilent Technologies, Santa Clara, CA, USA, 2011). Data was presented by total ion chromatogram (TIC). Minimum absolute abundance was set at 1000. The identification of the metabolites was done by using Mass Profile Professional (MPP) Software version B.12.01 (Agilent Technologies, Santa Clara, CA, USA). The metabolites that could not be identified were excluded from further analysis while the identified ones were collected and processed for further recursive analysis. Further analysis was done by analysis of variance (ANOVA), principle component analysis (PCA), and clustering test.

Data mining was performed by the molecular feature extractor (MFE) algorithm in the Agilent Mass Hunter Workstation Software B.07.00 (Agilent Technologies, Santa Clara, CA, USA, 2013). Noise was removed by using relative height parameter which was set at 1% of largest peak. The setting was applied for data processing method and used to process all generated data files in batch mode. Compound exchange format file was created for each sample and subjected to further data filtering and statistical analysis by MPP. Each sample was analyzed in triplicate with four biological replicates.

The filter (frequency analysis) was performed to determine the compounds that were present 100% of the time in at least one studied group. Filtering by ANOVA was the next step in selecting entities that are of significant values. In order to identify metabolites with differences in abundance between the experimental groups, data was filtered using fold change analysis. Fold change of two was used to eliminate any possible discriminating compounds. Data recursion for the re-examination of data was automatically re-extracted to generate extracted ion chromatograms (EICs) using the software, followed by peak inspection on resulted EICs to eliminate false positive and false negative results. Finally, the confirmed metabolites were then statistically analyzed by PCA. Compounds which distinguished the experimental groups were selected by frequency filtering, ANOVA, and fold change analysis which were then qualified by recursion analysis. 

## 3. Results 

### 3.1. LC/MS Analysis

The chromatogram of LC/MS Q-TOF for global metabolomics analysis from ranibizumab-treated HTF and control groups is shown in Supplementary Figure S2. Before any filtering or testing was done, 851 metabolites were detected from the data. These include endogenous, exogenous, identified, and nonidentified small molecules. The metabolites represent a wide variety of groups such as fatty acids, amino acids, and peptides. They are involved in multiple biochemical reactions, especially in energy and lipid metabolism [45,46]. 

### 3.2. Metabolic Profiling Using Mass Profile Professional (MPP) Software

The MPP software allows comparison of differential expression of metabolites between different groups through a number of clustering methods including hierarchical clustering as shown in Supplementary Figure S3. In this study, ranibizumab-treated HTFs group showed marked differences compared to the control group.

As mentioned earlier, preliminary data showed 851 identified and nonidentified metabolites in both control HTFs and ranibizumab-treated HTF groups. However, the nonidentified metabolites were excluded from further analysis. Finally, statistical analysis at *p*-value of < 0.01 with cut off value of two-fold change showed that 31 metabolites were significantly upregulated in ranibizumab-treated HTFs group compared to HTFs control group (Table 1). Meanwhile, 123 metabolites were significantly downregulated in ranibizumab-treated HTFs group compared to HTFs control group as listed in Table 2. The principle component analysis (PCA) showed that metabolites from control groups and ranibizumab-treated groups are clearly separated and differently expressed, as shown in Supplementary Figure S4. 

From the upregulated list, six metabolites were identified as the most upregulated metabolites in ranibizumab-treated-HTFs, as shown in Table 3. However, none of the profiled metabolites have a noteworthy role in the fibroblast cell cycle or wound healing regulation. On the other hand, seven metabolites from the downregulated list have been identified to play significant role in cell proliferation and growth (Table 4). Among the selected markers, we found that they are worthy for further investigation. Therefore, we would like to discuss their roles in wound healing and fibroblast cell cycle in association with ranibizumab treatment.

## 4. Discussion

This study was driven by the needs to understand the mechanism of ranibizumab in regulating the wound healing process following trabeculectomy. Wound healing is known as a complex cascade which comprises four major steps: coagulation, inflammation, proliferation, and remodeling phases [47]. It involves a number of growth factors, cytokines, and chemokines, and due to its complexity and prolonged process, wound healing provides many target sites for possible wound modulation. Due to the renowned properties of ranibizumab in minimizing scar formation at surgical site, we explored the antifibrotic effects of ranibizumab on cultured HTFs at the metabolite level.

In this study, we have identified many upregulated and downregulated metabolites in ranibizumab-treated HTFs. However, we focused on the most downregulated metabolites which are related to cell cycle and proliferation. Spermidine, a family member of polyamine, was implicated as the most significantly downregulated metabolite in ranibizumab-treated HTFs. It is known to have pleiotropic effects on cell physiology and plays a significant role in cell differentiation and proliferation, particularly in fibroblasts [48,49]. Synthesis of spermidine is enhanced in cells undergoing rapid growth activity as demonstrated in rat liver, mouse parotid gland, human diploid fibroblasts, and 3T3 mouse fibroblasts [50,51,52]. Disruption in spermidine level will lead to total inhibition of protein synthesis and growth arrest, as reported in HeLa cells [53]. Additionally, Rupniak et al. has reported that the inhibition of spermidine synthesis by methylglyoxal-bis (guany1hydrazone) (MGBG) led to growth arrest at G1 phase in rat embryo fibroblast (REF) [50]. The level of arginine, a precursor amino acid involved in spermidine synthesis [54], was also found to be significantly lower in the ranibizumab-treated HTFs group than in the control group. Both arginine and spermidine have been pointed as important components in polyamine biosynthesis. The conversion of arginine to ornithine by arginase initiates the polyamine metabolic pathway. Subsequently, the biosynthesis of spermidine is mediated by the key enzyme ornithine decarboxylase (ODC) which converts ornithine to putrescine and subsequently spermidine and spermine in the presence of N1-acetylspermidine and N1-acetylspermin enzymes [55,56,57].

In the current study, the level of pantothenic acid, D-Biotin, and folic acid were lower in ranibizumab-treated HTF groups than in the control groups. These vitamin B complex derivatives are important in the wound healing process. They act as a cofactor in a numbers of enzyme systems and their absence may disrupt normal protein, carbohydrate, and fat metabolism. Biotin is one of the best studied vitamins related to mitochondrial function and its deficiency in human may impair mitochondrial function and accelerate oxidative stress formation and the aging process [58,59,60]. Depletion in biotin inhibits heme synthesis and impairs mitochondrial function in human lung fibroblasts, as reported by Atamma et al. [61]. In addition, deprivation of pantothenic acid and folic acid have been reported to cause a decrease in white cell function, which may have deleterious effects on the human wound healing process, as reported by Koros et al. [62].

Certain amino acids are particularly important in wound healing. Cystein is known to be a critical component of terminal peptide where it functions as a cofactor for enzyme processes in the formation of intracellular collagen molecules. Cysteine is biosynthesized from methionine in a process called transulfuration, where homocysteine is catalyzed to form cystathionine. Cystathionine is then cleaved by γ-cystathionase to produce cysteine. Therefore, it can be concluded that the level of cysteine is the best in representing the homocysteine metabolic rate [63]. Cysteine-rich domains of Muc3 mucin stimulate cell migration in vitro and accelerate wound healing in vivo and vice versa, as demonstrated by Ho et al. [64]. C16 Sphinganine is one of the intermediate molecules in ceramide metabolism. Ceramide is the bioactive sphingolipids that regulates many cellular activities, including apoptosis, proliferation, and differentiation [65,66]. Marked increase in lipid components, particularly ceramide, has been reported in progressive wound healing of dermal fibroblasts [67,68]. In fact, upon wound formation, the intracellular level of sphingolipid was increased by the activation of ceramide-generating enzymes which in turn increased the expression of perixosome proliferator-activated receptor-ß (PPAR-ß) and subsequently accelerated the wound healing processes [69,70,71]. Thus, suppression of lipid level may lead to a delay in wound closure [72].

The interrelation of the above metabolites is well understood. They are either partly or completely involved in the folic acid metabolic cycle, homocysteine metabolism, and spermidine metabolism, which have major influences on HTFs proliferation and survival rate. Our data clearly demonstrated that the addition of ranibizumab on cultured HTFs significantly reduced these metabolites’ expression. Disruption of any one metabolite will impair the interrelated metabolic chain, thus compromising the conventional fibrotic activity. This will eventually result in less extracellular matrix component accumulation, the hallmark of successful trabeculectomy. In addition, identification of these metabolites will lead to more comprehensive studies on the specific pathways involved in wound healing. Understanding of the actual anti-scarring mechanisms of ranibizumab will offer better approaches to wound modulation strategies and subsequently increase the success rate of trabeculectomy.

## 5. Conclusions

The properties of the metabolites discussed above correlate with our findings. Downregulation of the discussed metabolites reduces fibroblastic activity, serving the objectives of ranibizumab application. We speculate that ranibizumab abrogates the tissue scarring process and wound healing in HTFs by regulating the expression of several metabolites associated with fibrotic activity. More intensive studies on the protein and gene level will be carried out to understand the mechanism of action of ranibizumab as an antifibrotic agent in trabeculectomy.

## Figures and Tables

**Table 1:** List of Upregulated Metabolites in Ranibizumab-Treated HTFs Compared to Control HTFs. At a *p*-value < 0.01 with a cut off value of two-fold change, 31 metabolites were significantly upregulated in the ranibizumab-treated HTFs group compared to the HTFs control group.

Metabolites	*p* Value
Oleamide	2.38 × 10^−3^
Stigmatellin Y	1.25 × 10^−3^
Ethyl 2-benzylacetoacetate	8.84 × 10^−10^
(5R,6S)-5,6-Epoxy-7-megastigmen-9-one	1.11 × 10^−4^
Spiroxamine	1.16 × 10^−3^
Spirolide D	1.86 × 10^−22^
Pipericine	2.54 × 10^−3^
Diisobutyl phthalate	2.10 × 10^−3^
11-Hydroxyeicosatetraenoate glyceryl ester	3.50 × 10^−9^
Ginsenoyne K	1.88 × 10^−9^
Trimeprazine	2.86 × 10^−5^
Verimol C	1.70 × 10^−3^
6-Acetyl-2,2-dimethyl-2H-1-benzopyran	2.13 × 10^−3^
Milbemectin	3.63 × 10^−3^
1-Isopropyl-2-methylbenzene	5.03 x 10^−4^
Homodihydrocapsaicin	2.46 × 10^−5^
Artabsinolide A	2.52 × 10^−3^
Betavulgaroside I	4.05 × 10^−3^
Isolinderanolide	6.01 × 10^−3^
Serinyl-Proline	2.76 × 10^−3^
Cyclocalopin F	3.31 × 10^−4^
3-Methyl-4-phenyl-3-buten-2-one	4.42 × 10^−3^
6-Oxabicyclo[3 .1.0]hexane-2-undecanoic acid methyl ester	4.99 × 10^−3^
Butocarboxim	4.34 × 10^−3^
Dodecanamide	4.88 × 10^−3^
Dioscoretine	5.40 × 10^−3^
Linalyl hexanoate	5.20 × 10^−3^
Devapamil	6.05 × 10^−3^
1-Acetoxy-2-hydroxy-5,12,15-heneicosatrien-4-one	5.74 × 10^−3^
Dihydroceramide	3.64 × 10^−4^
Glycidyl oleate	5.66 × 10^−3^

**Table 2:** List of Downregulated Metabolites in Ranibizumab-Treated HTFs Compared to Control HTFs. At *p*-value < 0.01 with a cut off value of two-fold change, 123 metabolites were significantly downregulated in the ranibizumab-treated HTFs group compared to the HTFs control group.

Metabolites	*p* Value
Armillarin	9.31 × 10^−6^
Polyoxyethylene (600) mono- ricinoleate	5.04 × 10^−8^
Nordihydrocapsaicin	2.94 × 10^−8^
C16 Sphinganine	1.73 × 10^−5^
Ergotamine	1.32 × 10^−4^
Phosphohydroxypyruvic acid	5.98 × 10^−4^
Marasmene	4.15 × 10^−7^
Stearic acid	2.88 × 10^−7^
Dioctyl hexanedioate	2.32 × 10^−3^
Thermospermine	1.24 × 10^−6^
Dodecyl butyrate	2.86 × 10^−7^
Alpha-Methylstyrene	1.54 × 10^−8^
Kanzonol K	4.32 × 10^−6^
Pirimicarb	8.14 × 10^−5^
Bryotoxin A	2.32 × 10^−6^
D-Quinovose	2.15 × 10^−7^
2-N-Undecyltetrahydrothiophene	1.74 × 10^−6^
Bakkenolide D	2.95 × 10^−7^
Auriculoside	1.53 × 10^−7^
Crassostrea Secocarotenoid	3.98 × 10^−4^
Swietenidin B	3.35 × 10^−6^
1-Aminocyclohexanecarboxylic acid	1.63 × 10^−5^
Netilmicin	5.59 × 10^−4^
Phytosphingosine	5.68 × 10^−07^
Cucurbitacin S	7.96 × 10^−4^
Epoxyeremopetasinorol	3.72 × 10^−7^
2-Methylbutyrylglycine	1.02 × 10^-5^
8-beta-Angeloyloxy-15-hydroxy-1alpha,10R-dimethoxy-3-oxo-11(13)-germacren-12,6alpha-olide	1.16 × 10^−3^
Octylamine	7.76 × 10^−4^
9,10-12,13-Diepoxyoctadecanoate	9.23 × 10^−6^
(3-Methylcrotonyl)glycine methyl ester	4.59 × 10^−6^
4-Ethylbenzaldehyde	2.84 × 10^−6^
Chenodeoxycholic acid sulfate	1.21 × 10^−3^
Istamycin C1	5.64 × 10^−4^
Acetyl tributyl citrate	5.24 × 10^−4^
3-Hydroxy-1-methylestra-1,3,5(10),6-tetraen-17-one	8.15 × 10^−4^
cis-3-Hexenyl trans-2-hexenoate	2.30 × 10^−6^
Piperolein B	4.29 × 10^−4^
Hexazinone	3.57 × 10^−6^

**Table 3:** List of Most Upregulated Metabolites in HTFs-Treated Group. From the upregulated list, six metabolites were identified as the most upregulated metabolites in ranibizumab-treated-HTFs.

Metabolites	*p*-Value
Ethyl 2-benzylacetoacetate	8.84 × 10^−10^
Spirolide	1.86 × 10^−22^
11-Hydroxyeicosatetraenoate glyceryl ester	3.50 × 10^−9^
Ginsenoyne K	1.88 × 10^−9^
Trimeprazine	2.86 × 10^−5^
Homodihydrocapsaicin	2.46 × 10^−5^

**Table 4:** List of Most Downregulated Metabolites in HTFs-Treated Group. From the downregulated list, seven metabolites were identified as the most downregulated metabolites in ranibizumab-treated HTFs.

Metabolites	*p*-Value
Pantothenic acid	7.33 × 10^−5^
Folic acid	8.28 × 10^−5^
Arginine	2.18 × 10^−5^
Cysteine	9.74 × 10^−5^
Spermidine	9.97 × 10^−6^
C16 Sphinganine	1.73 × 10^−5^
D-Biotin	8.73 × 10^−5^

## Supplementary Materials

The following are available online at https://www.mdpi.com/2218-273X/9/6/243/s1, Figure S1: Immunostaining Images of HTF By Vimentin And DAPI. A: Cytoplasm Stained in Green (Vimentin). B: Nucleus Stained in Blue (DAPI). C: Merge. D: Monochrome. (20x magnification). Figure S2: Chromatogram of LC MS Q-TOF Analysis. Figure S3: Hierarchical Clustering (HCA) view showing differences between control HTF groups to ranibizumab-treated HTF group. Figure S4: Principle component analysis (PCA) of metabolites from HTF control groups and ranibizumab-treated HTF group.
